# Supplementary figures and images for: Temporal fitness fluctuations in experimental Arabidopsis thaliana populations
Source: PLoS One. 2017 Jun 12;12(6):e0178990. doi: 10.1371/journal.pone.0178990 (PMC5467858; doi:10.1371/journal.pone.0178990)

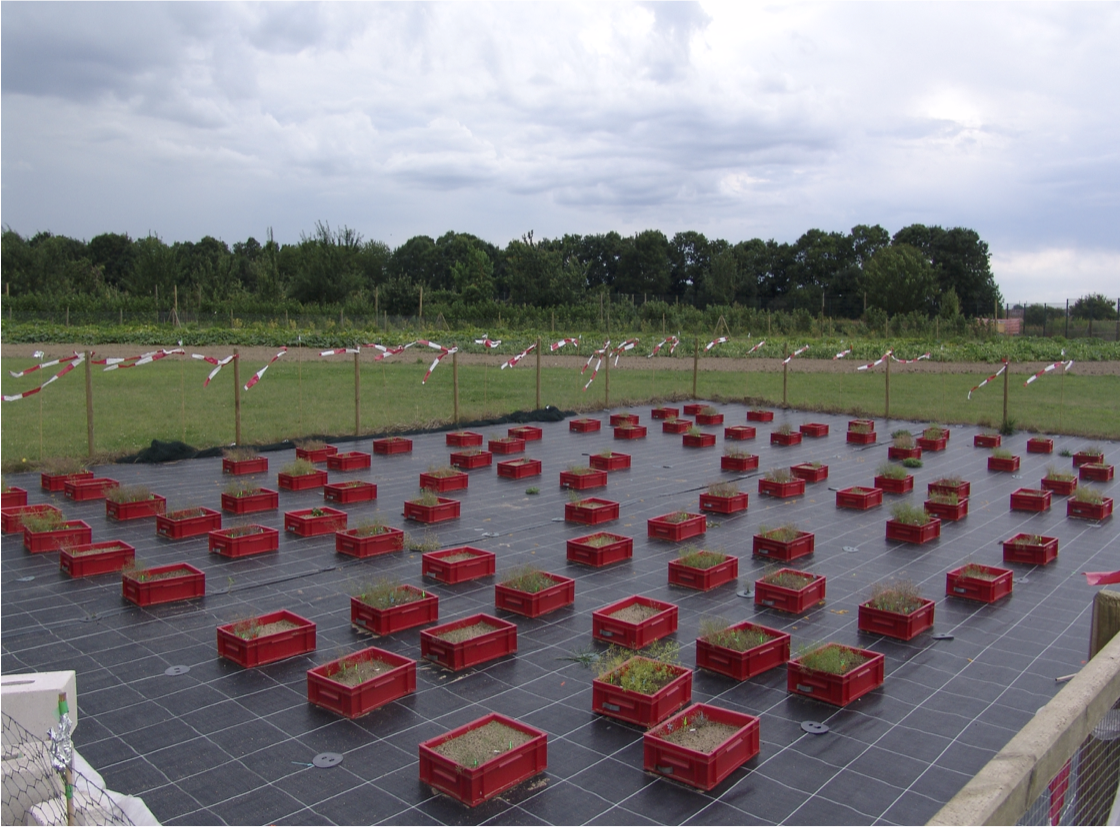

Supplement: S1 Fig — (TIFF) [file pone.0178990.s001.tiff]
